# Auto-regulatory feedback by RNA-binding proteins

**DOI:** 10.1093/jmcb/mjz043

**Published:** 2019-05-08

**Authors:** Michaela Müller-McNicoll, Oliver Rossbach, Jingyi Hui, Jan Medenbach

**Affiliations:** 1 Institute of Cell Biology and Neuroscience, Goethe University Frankfurt, Max-von-Laue-Strasse 13, D-60438 Frankfurt am Main, Germany; 2 Institute of Biochemistry, Justus-Liebig-University Giessen, Heinrich-Buff-Ring 17, D-35392 Giessen, Germany; 3 State Key Laboratory of Molecular Biology, CAS Center for Excellence in Molecular Cell Science, Shanghai Institute of Biochemistry and Cell Biology, Chinese Academy of Sciences, Shanghai 200031, China; 4 Institute of Biochemistry I, University of Regensburg, Universitaetsstrasse 31, D-93053 Regensburg, Germany

**Keywords:** autogenous regulation, protein homeostasis, RNA-binding proteins, post-transcriptional regulation of gene expression

## Abstract

RNA-binding proteins (RBPs) are key regulators in post-transcriptional control of gene expression. Mutations that alter their activity or abundance have been implicated in numerous diseases such as neurodegenerative disorders and various types of cancer. This highlights the importance of RBP proteostasis and the necessity to tightly control the expression levels and activities of RBPs. In many cases, RBPs engage in an auto-regulatory feedback by directly binding to and influencing the fate of their own mRNAs, exerting control over their own expression. For this feedback control, RBPs employ a variety of mechanisms operating at all levels of post-transcriptional regulation of gene expression. Here we review RBP-mediated autogenous feedback regulation that either serves to maintain protein abundance within a physiological range (by negative feedback) or generates binary, genetic on/off switches important for e.g. cell fate decisions (by positive feedback).

## Introduction

Post-transcriptional regulation (PTR) of gene expression plays an essential role in all eukaryotic cells. It allows the dynamic and rapid control of protein synthesis to adapt to the cellular requirements, e.g. during differentiation or in changing environments. PTR is mostly governed by RNA-binding proteins (RBPs) that associate with *cis*-acting RNA elements to control all aspects of RNA metabolism from synthesis to decay.

Recent methodological advances have greatly expanded the number of identified RBPs. These studies demonstrated that RBPs are found among a broad spectrum of protein families involved in diverse biological processes, some of them even exhibiting enzymatic activities in cellular metabolism. This intimate connection of RNA biology with in principle unrelated processes such as intermediary metabolism provides further evidence for a central role of RNA-based gene regulation in eukaryotic organisms (Castello et al., 2015; Beckmann et al., 2016).

Given the importance of RBPs for the regulation of RNA metabolism, it is not surprising that their expression levels need to be tightly controlled. Both overproduction as well as failure to synthesize sufficient amounts of a given RBP can have deleterious consequences. Here we discuss the diverse auto-regulatory circuits that RBPs employ to maintain protein homeostasis (by negative feedback) or to generate binary, genetic switches that govern cell fate decisions (by positive feedback).

Auto-regulation by RBPs has been discovered more than four decades ago in bacteria infected with bacteriophage T4 (Russel et al., 1976) and, subsequently, similar regulatory circuits were identified in archaea and eukaryotes. Meanwhile, autogenous regulation is considered to be an important mechanism of PTR and numerous human RBPs are proposed to engage in direct auto-regulatory feedback (Zanzoni et al., 2013).

Given the wealth of examples of auto-regulation found among RBPs, we cannot comprehensively cover the topic in this review (we apologize to all colleagues whose work we may not cite). Rather, we aim to provide an overview of the broad spectrum of auto-regulatory pathways that RBPs employ to control their own production, focusing on select examples of feedback regulation where the proteins directly associate with their own transcripts. Circuits where the regulatory RBPs control their own synthesis indirectly, e.g. through fidelity of initiation codon recognition during translation initiation (Ivanov et al., 2010; Loughran et al., 2012), via miRNA processing (Ratnadiwakara et al., 2018), by affecting the levels of spliceosomal ribonucleoproteins (RNPs; Jodelka et al., 2010), or where association with the RNA is mediated by adapter proteins (Rouhana and Wickens, 2007) will not be addressed. Moreover, we exclusively discuss regulation in eukaryotic organisms—for RBP-mediated auto-regulation in prokaryotes, please refer to other reviews (Betney et al., 2010; Meyer, 2018).

## Importance of RBP abundance: the right dose differentiates a poison from a remedy

Given that many RBPs perform important cellular functions, their loss or even reduced levels can result in strong cellular phenotypes, reduced cellular fitness, or cell death. Moreover, mutations in RBP-encoding genes are linked to numerous diseases spanning a broad spectrum of pathologies from neurological disorders to various types of cancer (Castello et al., 2013).

Overexpression of RBPs also results in aberrant gene expression and can have an equally deleterious effect on cellular fitness. Apart from outcompeting other co-regulatory RBPs on shared target sites, the increased abundance of a given RBP changes the rate constant by which it associates with its RNA target sites and, by mass action, permits binding to low-affinity RNA sites that does not occur at physiological protein concentrations (Darnell, 2010; Wright et al., 2011; Riley and Steitz, 2013). Excess protein can therefore promote adventitious regulation of ‘non-physiological’ targets, which results in neomorphic activity. Thus, even subtle changes to auto-regulatory feedback regulation can compromise cellular fitness (Li et al., 1996), underscoring the importance to maintain RBP levels and function in a narrow physiological range to prevent non-specific binding and mis-regulation. Moreover, it has been observed that several RBPs that elicit autogenous regulation contain aggregation-prone disordered regions, e.g. TAR DNA-binding protein 43 (TDP43) and fused in sarcoma (FUS), suggesting that auto-regulation also plays an important role in preventing the formation and accumulation of toxic RBP aggregates (Zanzoni et al., 2013; Weskamp and Barmada, 2018).

## RBPs exert feedback regulation at different post-transcriptional levels

RBPs can control gene expression at different steps from RNA synthesis to its decay. Often the mechanism by which RBPs control their own levels is the same by which they regulate their target RNAs. For example, splicing factors such as SR proteins or heterogeneous nuclear RNPs (hnRNPs) mediate unproductive alternative splicing (AS) of their mRNAs upon overexpression. This generates transcripts that contain premature termination codons (PTCs), which trigger rapid RNA degradation (Wollerton et al., 2004; Lareau et al., 2007; Ni et al., 2007). Other splicing factors, such as the Fox proteins, use AS to produce dominant negative isoforms that compete with the full-length proteins (Damianov and Black, 2010). Translational regulators, such as SRSF1, instead inhibit the translation of their own mRNAs (Sun et al., 2010), while the export factor NXF1 binds and promotes the export of its own transcript that contains a retained intron, which leads to either to rapid degradation of this RNA or to synthesis of a truncated, inactive NXF1 protein isoform (Li et al., 2006).

In some cases, RBPs exert homeotic feedback at multiple levels. The yeast ribosomal protein L32 for example can control both splicing and translation of its own mRNA, matching its production to the synthesis rate of ribosome precursors (Dabeva and Warner, 1993). The *Drosophila* protein Sex-lethal (Sxl) even employs both positive and negative feedback mechanisms that operate at the level of splicing and translation. This results in binary switch-like gene expression (mediated by positive feedback), while simultaneously preventing deleterious overproduction of the protein by negative feedback (Moschall et al., 2017).

## Homeostatic feedback regulation

Maintenance of proteostasis is especially challenging when considering different cell sizes or cellular growth: while the volume of the cytoplasm (and the nucleus) changes, the DNA content remains constant. In order to maintain physiological protein concentrations, cell growth therefore necessitates an increased number of protein molecules from a fixed number of alleles. An elegant way to control RBP abundance and to dynamically adjust protein concentrations, e.g. during cell growth, is auto-regulatory, negative feedback where the proteins exert homeostatic control over their own production. This homeostatic feedback functions as a built-in adaption mechanism that automatically buffers against changes in cellular protein levels stemming for example from heterozygosity or from fluctuations inherent to gene expression (Becskei and Serrano, 2000; Freeman, 2000; Swain, 2004), providing robustness to the steady-state levels of RBPs.

**Figure 1:**
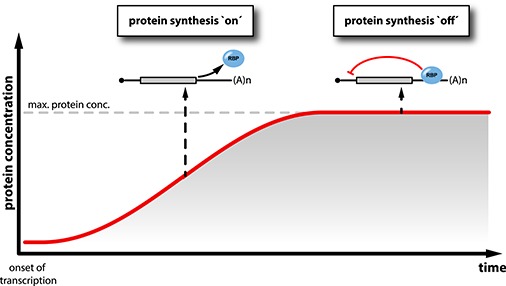
Auto-regulatory negative feedback limits protein accumulation. Schematic representation of protein abundance as a function of time after transcriptional induction of a gene that encodes an RBP with auto-regulatory activity. At low protein concentrations, the RBP cannot engage in feedback regulation and protein synthesis ensues (left). After accumulation for a higher protein concentration, negative feedback is triggered: the protein binds to regulatory sequences present in its own mRNA and exerts its auto-regulatory activity (e.g. translational repression, schematically pictured on the right), thus limiting further protein synthesis.

Physical interaction of a given RBP with its own transcript is primarily not only a function of protein concentration but also depends on (i) its sub-cellular localization, which affects local protein concentration, (ii) biophysical parameters of the interaction, e.g. affinity to the binding site, stoichiometry and dynamics of the interaction, abundance of competing RNA sequences, and (iii) other factors that impact on protein activity, such as inhibitors or competitors, the requirement for additional co-regulatory factors, or post-translational modifications. If the protein concentration is low and/or if its RNA-binding activity is compromised, it does not bind to the regulatory RNA sequences present in its own transcript. It therefore cannot exert its auto-regulatory activity and protein production ensues. However, once a critical concentration of the RBP is reached (usually in the range of its dissociation constant (K_D_) with the RNA) and once the RNA-binding activity of the protein exceeds a certain threshold, it engages in interactions with the mRNA to repress protein production (Figure 1). Auto-regulation therefore directly reads out RNA-binding activity of the RBP, which only indirectly depends on parameters such as transcriptional activity or cell size.

This type of auto-regulation also implies that, in the absence of transcriptional regulation, the steady-state concentration of the RBP primarily depends on the strength of the interaction with its own transcript. If regulation occurs via high-affinity binding sites, protein accumulation is already attenuated at low concentrations. In contrast, low-affinity binding sites allow for a much higher steady-state protein level to be reached, before negative feedback is triggered (Stapleton et al., 2012). The final protein concentration therefore appears to be—at least partially—genetically hard-wired. This suggests an intimate co-evolution of the RBP and RNA binding site(s) present within its own mRNA: the affinity for their own transcript should lie right in between the affinities for the target RNAs to be regulated and the ‘non-targets’ that carry low affinity sites that can mediate adventitious regulation. This is also reflected by the high evolutionary conservation of the regions containing these binding sites (Lareau et al., 2007).

Although the steady-state concentrations of auto-regulatory proteins appear to be to a certain extent genetically fixed, dynamic regulation of protein abundance can still be achieved. For example, post-translational modification of RBPs within their RNA-binding domains (RBDs; such as methylation or acetylation) as well as modifications of its RNA binding site in response to signaling (Blee et al., 2015; Adhikari et al., 2016) can impact on the auto-regulatory feedback and affect the protein steady-state level. Similarly, altered sub-cellular localization of the RBP can impact on its auto-regulatory activity and its abundance (Yi et al., 2010; Kotta-Loizou et al., 2014; Fagg et al., 2017; Weskamp and Barmada, 2018). Moreover, in many cases auto-regulatory pathways require additional co-regulators (Suissa et al., 2011; Kolesnikova et al., 2013), or inhibitory factors can antagonize autogenous feedback, e.g. by competition for the same RNA elements (Moschall et al., 2018). Control of the abundance or the activity of these co-factors or inhibitors provides an additional mechanism to adjust and fine-tune the steady-state levels of auto-regulatory RBPs.

### Unproductive AS

**Figure 2:**
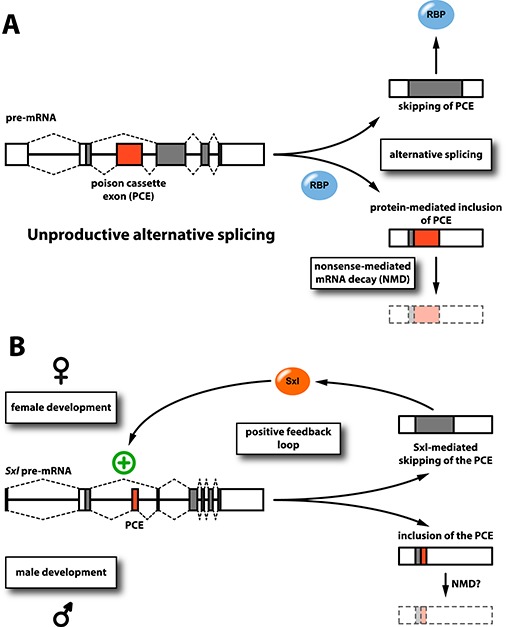
Common principles in feedback regulation to splicing. (**A**) Auto-regulation by unproductive AS creates a negative feedback loop. Auto-regulatory feedback is exerted by control of the inclusion of a PCE (highlighted in red) that contains a PTC. By default, splicing of the pre-mRNA (schematically depicted on the left) results in skipping of the PCE, generating an mRNA that encodes functional protein (depicted at the top). Once a critical concentration of the RBP is produced, it limits its own synthesis by promoting inclusion of the PCE during splicing (depicted below). This results in the generation of mRNAs with a shortened open reading frame that encodes a truncated and non-functional protein isoform. The presence of the PTC can furthermore trigger rapid mRNA destabilization and turnover via the NMD pathway. (**B**) Sxl auto-regulatory, positive feedback to AS generates a molecular switch that controls sexual development. *Drosophila* Sxl acts as a molecular switch that controls female development. Once produced, Sxl protein engages in an auto-regulatory positive feedback loop promoting skipping of a PCE in its own transcript (depicted at the top). This ensures lasting Sxl protein production and governs female development. Male development is characterized by the absence of functional Sxl protein and inclusion of the PCE during splicing. This generates mRNAs that encode a truncated and non-functional protein isoform and are likely degraded by the NMD pathway (depicted at the bottom). Exons are depicted as boxes, introns in the pre-mRNAs as lines. The AS patterns are indicated by dashed lines in the pre-mRNAs, open reading frames in the mature mRNAs (on the right of each panel) are shaded grey, and the PCEs are highlighted in red.

Many RBPs, among them splicing factors of the hnRNP and SR protein families, employ negative feedback loops to auto-regulate their protein levels. One of the most prevalent mechanisms for such regulatory feedback is AS-coupled nonsense-mediated mRNA decay (AS-NMD; Ottens and Gehring, 2016), which is also known as regulated unproductive splicing and translation (RUST; Lareau et al., 2007) (Figure 2A). The NMD pathway is a cytoplasmic, translation-dependent surveillance mechanism that degrades transcripts with PTCs (Fatscher et al., 2015). Such PTCs are frequently introduced by AS, resulting in rapid turnover of the transcripts (Lewis et al., 2003).

SR proteins are a family of essential RBPs that regulate constitutive and AS in all metazoan cells. Through their additional pre- and post-splicing activities, they are important players in connecting nuclear and cytoplasmic steps of gene expression (Müller-McNicoll et al., 2016). In mammals, the SR protein family comprises 12 canonical members (SRSF1–SRSF12) that share a common domain structure with one or two RBDs and an arginine and serine (RS) domain of different length, which consists mainly of RS repeats. SR proteins act mostly as splicing activators. They bind to exonic or intronic splicing enhancer elements (ESEs, ISEs) within pre-mRNAs and use their RS domain as protein-interaction platform to promote the recruitment of the splicing machinery to neighboring splice sites.

Although SR proteins are very abundant RBPs, different family members are expressed in a tissue-specific manner and overall their expression levels are tightly controlled. Perturbations in SR protein levels change the AS pattern of pre-mRNAs dramatically and are associated with numerous diseases such as cancer, systemic lupus erythematosus (SLE) or spinal muscular atrophy (SMA; Shilo et al., 2015). For example, SRSF1–SRSF7 are considered proto-oncogenes exhibiting abnormal expression in many tumors and overexpression of SRSF1 is sufficient to transform fibroblasts, which then rapidly generate tumors in mice (Karni et al., 2007; Kedzierska and Piekielko-Witkowska, 2017).

Numerous SR proteins keep their protein levels constant by engaging in auto-regulatory feedback via AS-NMD (Risso et al., 2012). SR proteins employ three different mechanisms for the generation of NMD-sensitive transcript isoforms. SRSF3–SRSF6, SRSF7, SRSF9, and SRSF10 promote the inclusion of an ultra-conserved alternative exon that contains a PTC. This poison cassette exon (PCE) then triggers RNA degradation via the NMD pathway. Although a strict auto-regulation via PCE inclusion was only demonstrated for SRSF3 in human cells (Jumaa and Nielsen, 2000) and tra-2 in *Drosophila* (McGuffin et al., 1998), it has been hypothesized that similar feedback loops are employed by other SR proteins containing ultra-conserved exons (Lareau et al., 2007). SRSF1 and 2 instead appear to promote splicing of introns located within their 3′UTRs, which deposits exon-junction complexes (EJCs) downstream of the normal termination codons, thereby transforming the mRNAs into NMD targets (Sureau et al., 2001; Sun et al., 2010). In addition, SRSF5 prevents splicing of its entire intron 5 (surrounding the PCE), which also introduces PTCs and results in a potential NMD target (Lareau and Brenner, 2015).

The contribution of AS-NMD to the overall auto-regulation of SR protein levels remains to be investigated for most SR proteins. For example, AS-NMD alone is not sufficient to maintain homeostasis of SRSF1. A more complex auto-regulatory feedback loop comprising several control layers is required, including alternative polyadenylation (APA), translational repression, and mRNA destabilization via miRNAs (Sun et al., 2010).

HnRNPs are another class of RBPs that associate with nascent and mature transcripts and determine their fate. HnRNPs are the most abundant proteins in the nucleus (Dreyfuss et al., 2002). At least 37 hnRNP genes have been identified in the human genome, which are grouped into distinct subfamilies (Busch and Hertel, 2012). All hnRNP proteins share a common domain structure containing at least one RNA-binding domain, mainly of the RNA recognition motif (RRM) type, and auxiliary domains with clusters rich in certain amino acids.

Similarly to SR proteins, many hnRNPs engage in direct homeostatic feedback via AS-NMD. This was first reported for the polypyrimidine tract-binding protein (PTB, hnRNP I, PTBP1), which binds to its own pre-mRNA to repress inclusion of exon 11. This results in a frameshift, which generates a PTC in the subsequent exon and targets the RNA for NMD (Wollerton et al., 2004). PTB belongs to a family of closely related hnRNP proteins that comprise three members (PTBP1–PTBP3), which are expressed in a tissue-specific manner. PTBP2 (nPTB) is mainly expressed in neurons, whereas PTBP3 (ROD1) is expressed in hematopoietic cells. In addition to its auto-regulatory activity, PTB also negatively regulates the expression of its two paralogs by AS-NMD. Upon upregulation, nPTB can compensate for PTB in AS of several target pre-mRNAs, suggesting a functionally redundant but tissue-specific function. Both PTB and nPTB promote the nonproductive splicing of the third paralog ROD1. In all three pre-mRNAs, highly conserved regions have been associated with these AS events (Spellman et al., 2007).

Other RBPs that engage in auto- and cross-regulation between closely related paralogs are hnRNP L and L-like (LL; Rossbach et al., 2009) as well as hnRNP D (AUF1) and hnRNP D-like (DL; Kemmerer et al., 2018). HnRNP L exerts autogenous feedback regulation by binding to an unusually long and highly conserved intronic splicing enhancer element present within its pre-mRNA. The regulatory region contains a short PCE that, due to its destabilizing effect, is typically not detected in mature RNAs. When hnRNP L levels are high, inclusion of this PCE is increased, concomitantly reducing hnRNP L protein production. HnRNP L down-regulates its paralog hnRNP LL by a similar mechanism (Rossbach et al., 2009). HnRNP LL also exhibits tissue-specific expression and functions, and the interplay between hnRNP L and LL is crucial for the regulation of AS events during B-cell and T-cell activation (Preussner et al., 2012). Auto- and cross-regulation of hnRNP D and DL occur in a fashion analogous to hnRNP L and LL (Kemmerer et al., 2018).

Another intricate mechanism of auto-regulation is employed by the RNA-specific adenosine to inosine (A-to-I) editing enzyme ADAR. In rodents, ADAR2 binds to its own pre-mRNA and generates a novel splice acceptor site by A-to-I editing whose usage generates a transcript with a frameshift that encodes a truncated ADAR isoform with reduced enzymatic activity (Rueter et al., 1999). *Drosophila* ADAR (dAdar) can also edit its own mRNA. In contrast to the rodent system, however, this does not generate a new splice site but results in an amino acid substitution in the C-terminal catalytic domain of the encoded dAdar protein, reducing its activity (Palladino et al., 2000).

Auto-regulatory control of splicing is also observed for non-splicing factor RBPs, e.g. for the budding yeast RNA export factor Yra1 (Preker and Guthrie, 2006), the poly(A)-binding protein nuclear 1 (PABPN1; Bergeron et al., 2015), and various ribosomal proteins in different organisms such as yeast Rps9, Rpl22, and Rpl32 (Eng and Warner, 1991; Plocik and Guthrie, 2012; Gabunilas and Chanfreau, 2016), *C. elegans* L10a and L12 (Mitrovich and Anderson, 2000; Takei et al., 2016), *X. laevis* Rpl1 (Bozzoni et al., 1984), and the human ribosomal proteins S13 and L3 (Cuccurese et al., 2005; Malygin et al., 2007).

### Control of RNA 3′ end processing

Auto-regulation can also occur at the level of 3′ end processing and polyadenylation. For example, U1A protein, which primarily functions as a component of the U1 small nuclear RNP (U1 snRNP) during splicing, auto-regulates its protein levels through an intricate mechanism that prevents productive 3′ end processing and polyadenylation (Boelens et al., 1993). Upon U1A accumulation, two molecules of free U1A protein bind cooperatively to a bipartite RNA element within the 3′UTR of their own mRNA, which is located at a conserved distance to the polyadenylation site. This element contains a conserved secondary structure and is termed the polyadenylation inhibition element (PIE). Regulation requires the direct interaction of two molecules of U1A with the C-terminus of the poly(A) polymerase (PAP), the enzyme that generates the poly(A) tails, which inhibits its enzymatic activity (Gunderson et al., 1994, 1997; Varani et al., 2000). Functional PAP inhibitory motifs have also been identified in other RBPs such as U1-70k, SRSF4, and U2AF65, but it remains to be shown whether they engage in auto-regulatory feedback (Ko and Gunderson, 2002).

Another recent example is the cleavage and polyadenylation (CPA) factor PCF11, which auto-regulates its own protein level via premature polyadenylation and termination of transcription. This negative feedback loop is essential for normal development, and it was shown in zebrafish, mouse and human cells that, upon overexpression, PCF11 binds to a poly(A) site close to its own promoter to activate its usage. This causes premature polyadenylation and lowers the expression of functional *PCF11* transcripts (Kamieniarz-Gdula et al., 2019; Wang et al., 2019).

### Translational control

Auto-regulation at the level of translation is commonly observed and has been well studied for ribosomal proteins (r-proteins) in bacteria (recently reviewed in Meyer, 2018). Feedback regulation to translation by r-proteins is not only restricted to bacteria, but has also been detected in archaea (Daume et al., 2017) and, in isolated cases, in eukaryotes (Dabeva and Warner, 1993; Kim et al., 2010).

In eukaryotes also several enzymes of the intermediary metabolism are known to exhibit RNA-binding activity and to exert auto-regulatory feedback via attenuation of translation; the exact regulatory mechanisms, however, remain to be elucidated. Among the auto-regulatory enzymes are thymidylate synthase (TS; Chu et al., 1991; Liu et al., 2002), dihydrofolate reductase (DHFR; Ercikan et al., 1993), and serine hydroxymethyltransferase (SHMT; Liu et al., 2000) that function in one-carbon metabolism that is central to thymidine synthesis, amino acid homeostasis, and maintenance of the cellular redox status by keeping glutathione in a reduced state (Ducker and Rabinowitz, 2017). TS is a folate-dependent enzyme and important chemotherapeutic target in cancer therapy due to its enzymatic activity that generates dTMP to allow DNA synthesis for cellular proliferation. Its auto-regulatory activity is modulated by the redox status of the cell and by ligand binding (Chu et al., 1994). Similarly, the auto-regulatory inhibition of translation by DHFR is sensitive to dihydrofolate, its substrate, or the antifolate drug methotrexate that is employed in cancer therapy (Ercikan-Abali et al., 1997). For both enzymes, substrate binding suppresses feedback regulation, providing an elegant regulatory circuit to adjust protein levels to substrate availability.

Recent studies aimed at comprehensively identifying RBPs have provided evidence that many more enzymes of intermediary metabolism can associate with RNA (Castello et al., 2015; Beckmann et al., 2016). This suggests that post-transcriptional feedback regulation by metabolic enzymes might be more widely employed than previously anticipated, which has fueled the idea of regulatory networks based on RNA, enzyme, and metabolite interactions (the REM hypothesis; Hentze and Preiss, 2010).

### mRNA turnover

As described above, AS can generate PTC-containing mRNAs destined for rapid turnover by NMD. Auto-regulatory feedback to splicing often generates mRNAs with truncated open reading frames resulting from intron retention or inclusion of a PCE. These RNAs do not only trigger NMD but also, if translated, usually encode truncated and non-functional proteins (Ottens and Gehring, 2016). Moreover, incomplete processing of an mRNA (e.g. failure to generate a poly(A) tail) can result in nuclear retention of the transcript and its rapid turnover by nuclear RNA surveillance pathways (Schmid and Jensen, 2018).

Besides this ‘indirect’ effect on RNA stability by mis-processing or incomplete processing, there are also cases where auto-regulatory feedback directly induces RNA turnover. The yeast ribosomal protein Rps28 can associate with a conserved RNA hairpin structure present in the 3′UTR of its own mRNA and recruit the RNA decapping machinery through direct interaction with the enhancer of decapping 3 (Edc3) protein (Badis et al., 2004; Kolesnikova et al., 2013). Moreover, yeast Rpl4 was shown to induce endonucleolytic cleavage of its own transcript in the nucleus (Presutti et al., 1995).

The microprocessor is a protein complex comprising the RNaseIII enzyme DROSHA and the double-stranded RBP DGCR8. Microprocessor was shown to negatively regulate the expression of DGCR8 through cleaving a hairpin that is localized within the 5′UTR of *DGCR8* mRNA this way considerably enhancing its turnover (Triboulet et al., 2009). In addition to this, a miRNA is encoded within the *DROSHA* transcript, which upon processing by the microprocessor is able to attenuate expression of DROSHA protein (Mechtler et al., 2017).

The conserved hnRNP protein TDP43 also exerts negative feedback regulation by RNA destabilization (Ayala et al., 2011). It binds to GU-rich sequences present in the 3′UTR of its mRNA to promote RNA turnover, most probably via the exosome. Its auto-regulatory activity depends on a glycine-rich region in its C-terminus, which is also critical for its function in splicing, probably through interaction with hnRNP proteins. Cytoplasmic mis-localization and failure to engage in auto-regulatory feedback can result in accumulation and aggregation, which has been associated with numerous neurodegenerative disorders such as amyotropic lateral sclerosis (ALS; Weskamp and Barmada, 2018).

## Positive auto-regulatory feedback controls cell fate decisions

In contrast to negative feedback that serves homeostatic function, auto-regulatory positive feedback results in a switch-like gene expression pattern: a transient and often weak input signal is amplified and converted into a binary, all-or-nothing response. This is important, e.g. for generation of precise borders during pattern formation in embryonic development, as it conveys robustness to cell fate decisions (Perrimon et al., 2012).

### A Sxl feedback loop governs female development in Drosophila

Positive feedback regulation is exemplified by the RBP Sxl, which acts as the master regulator of female development in somatic tissues in *Drosophila*. It is expressed in a sex-specific fashion and exerts its feminizing activity by post-transcriptionally controlling the expression of key factors involved in sexually dimorphic traits (Moschall et al., 2017). Surprisingly, *Sxl* transcripts can also be detected in male flies. Inclusion of a PTC-containing PCE, however, prevents production of fully functional protein in males (similar to AS-NMD). To initiate female development, an X-chromosome counting mechanism produces a priming amount of Sxl protein early in embryonic development. At a later developmental stage, this protein then engages in an auto-regulatory feedback loop: it associates with its own primary transcript to suppress inclusion of the PCE, thereby promoting further expression of Sxl protein. This self-sustaining expression loop acts as a molecular switch that, once activated, ensures lasting Sxl protein expression, committing to female development (Figure 2B). Moreover, the positive feedback functions as a ‘cellular memory system’ and Sxl expression is inherited by daughter cells during mitosis (Salz and Erickson, 2010; Salz, 2011).

Despite the finding that the function of Sxl as master regulator of female development appears to be limited to only a few drosophilid species, similar concepts of sex determination and sexual development can be found in other insects where the SR protein *transformer* (and related RBPs) engage in auto-regulatory, positive feedback to promote female development (Salz, 2011; Sawanth et al., 2016).

Interestingly, Sxl does not only exert positive feedback by promoting ‘productive’ splicing of its own mRNA for its sustained expression. It also inhibits its own translation, exerting negative feedback to prevent accumulation of excessive Sxl protein levels (Yanowitz et al., 1999). Another abundant RBP of the hnRNP A/B family, Hrp48, has also been implicated in the homeostatic control of Sxl protein levels (Suissa et al., 2010). It has been proposed that, in order to exert its repressive activity on Sxl production, it requires the Sxl protein itself as a co-factor such that if Sxl levels become too low, the regulation is alleviated (Suissa et al., 2011). This provides an additional feedback mechanism to keep Sxl protein levels at physiological concentrations and to prevent deleterious overproduction.

### Auto-regulation by cytoplasmic polyadenylation

Positive auto-regulation was also demonstrated for the *Drosophila* oo18 RBP (Orb; Tan et al., 2001). It plays a critical role in numerous processes including memory formation, meiotic entry, egg chamber formation, and axis determination in the early embryo (Christerson and McKearin, 1994; Lantz et al., 1994; Huynh and St Johnston, 2000; Pai et al., 2013). For axis determination, it contributes to the localization and local translation of the *oskar* and *gurken* mRNAs, which encode critical determinants of anterior–posterior and dorsoventral polarity. The Orb protein shares homology with the vertebrate cytoplasmic polyadenylation element-binding proteins (CPEBs) involved in the regulation of protein synthesis via control of poly(A)-tail length (Mendez and Richter, 2001). It has been proposed that Orb-mediated translational activation of the *oskar* and *gurken* mRNAs, as well as its auto-regulatory activity, are mediated by cytoplasmic polyadenylation that enhances RNA stability and translation (Chang et al., 1999; Tan et al., 2001; Derrick and Weil, 2017).

Another case of auto-regulatory, positive feedback is found in meiotic progression during *Xenopus* oocyte maturation, which critically depends on the activity of *Musashi* hnRNP-type RBPs. *Musashi* proteins also participate in stem cell maintenance by suppressing the translation of mRNAs that encode proteins involved in cellular differentiation. Their function is conserved from flies to mammals and their elevated expression has been implicated in cancer development and maintenance of cancer stem cells (Kudinov et al., 2017). During oocyte maturation in *Xenopus*, they engage in auto-regulatory feedback to stimulate their own expression (Arumugam et al., 2012). For this, they associate with a *cis*-acting RNA regulatory element present in the 3′UTR of the *Musashi1* mRNA to trigger cytoplasmic polyadenylation, thereby increasing RNA stability and translation. Their stimulatory effect on cytoplasmic polyadenylation is due to the recruitment of the cytoplasmic PAP either via direct interaction (Cragle and MacNicol, 2014) or by modulating the association of CPEBs that in turn interact with the polymerase (Weill et al., 2017).

### HuR auto-regulation in replicative senescence and cancer

Human antigen R (HuR), a member of the embryonic lethal abnormal vision (ELAV) protein family, is another example of an RBP that engages in both positive and negative autogenous regulation. HuR can control different aspects of gene expression, including RNA processing, stability and translation, exerting its regulatory functions mostly through binding to AU-rich RNA sequence elements (AREs; Hinman and Lou, 2008). AREs can be detected in up to 8% of the human genes and they dynamically associate with a diverse group of proteins (ARE-RBPs), among them AU-binding factor (AUF1 aka hnRNP-D), tristetraprolin (TTP), and T-cell intracellular antigen 1 (TIA1). RNA binding of these proteins is often mutually exclusive and, depending on which set of proteins is associated with the ARE, the transcript is channeled into different pathways. For example, AUF1 and TTP binding usually trigger rapid RNA decay, while HuR can stabilize ARE-containing RNAs and promote their translation. Many ARE-RBPs associate with their own transcripts, suggesting that they might exert autogenous feedback regulation and, in addition, numerous cases of cross-regulation have been reported among them (Pullmann et al., 2007; Garcia-Maurino et al., 2017).

Several feedback circuits have been reported for HuR that operate based on the autogenous control of APA, RNA export, and stability (Al-Ahmadi et al., 2009; Yi et al., 2010; Dai et al., 2012). Association of HuR with elements in the 3′UTR of its own transcript can result in either increased protein expression based on increased stability, export, and translation of the RNA (Yi et al., 2010) or in attenuated protein production (Dai et al., 2012).

Homeotic auto-regulation of HuR is mediated by a GU-rich RNA element that overlaps with the major polyadenylation signal of the RNA. Association of HuR reduces the binding of a subunit of the cleavage stimulation factor (CstF-64) and shifts polyadenylation to a more distal site. This generates an RNA isoform with a longer 3′UTR carrying an ARE that promotes enhanced turnover of the RNA and reduces protein production (Dai et al., 2012).

The HuR protein levels need to be tightly controlled, as its over-expression contributes to pathology, e.g. during inflammation or in the formation of cancer and its progression (Wang et al., 2013; Kotta-Loizou et al., 2014; Shang and Zhao, 2017). Notably, the auto-regulatory feedback circuits employed by HuR appear to operate in different cellular compartments: negative auto-regulation requires nuclear HuR (Dai et al., 2012), while positive feedback regulation depends on its cytoplasmic localization (Yi et al., 2010). In line with this, inhibition of nuclear export by leptomycin B or knockdown of the nuclear export factor exportin-1 results in a significant reduction of HuR protein levels. A similar change in its nucleo-cytoplasmic distribution can be observed during replicative senescence, upon which the total HuR protein levels are reduced. Conversely, in several types of cancer, an increase in cytoplasmic HuR can be detected, which correlates with tumor progression and poor patient survival (Wang et al., 2013; Kotta-Loizou et al., 2014). As HuR controls the expression of many RNAs that encode cancer-relevant proteins, it is considered to play a central role in cancer biology. Altered nucleo-cytoplasmic distribution of HuR and concomitant changes to its feedback regulation might therefore play an important role in tumor formation and progression.

## Outlook

The previous examples underscore the importance of autogenous feedback by RBPs in the regulation of gene expression. Changes to feedback regulation of individual RBPs can upset cellular homeostasis and contribute to disease. While isolated cases of RBP-mediated auto-regulation have been well studied, in most cases, however, detailed mechanistic insight is lacking. A better understanding of the mechanisms underlying the auto-regulatory pathways (and their control) might pave the way for their therapeutic manipulation. Moreover, the simplicity and robustness of the auto-regulatory circuits make them particularly useful for a number of synthetic biology approaches, e.g. aimed at buffering against gene dose differences or at fine-tuning protein expression levels in mammalian cells (Stapleton et al., 2012; Mathur et al., 2017).

Furthermore, it remains to be determined how broadly autogenous regulation is employed by RBPs and how many of the newly discovered RBPs exhibit auto-regulatory activities. Their study might reveal yet additional feedback mechanisms or regulatory principles that are employed to provide robustness to gene expression and to maintain cellular homeostasis.

## Funding

This work was supported by the German Research Foundation (SFB902/2, B13 and CEF-MC to M.M.-M.; GRK 2355 to O.R.; SFB 960/2, B11 to J.M.), the German Federal Ministry of Education and Research (BMBF, 01ZX1401D to J.M.), the LOEWE program Medical RNomics (to O.R.), the National Natural Science Foundation of China (31570820, 31661143035, and 31770881 to J.H.), and the National Key Research and Development Program of China (2017YFA0504400 to J.H.).

**Conflict of interest:** none declared.
